# Seasonality effects on chemical composition, antibacterial activity and essential oil yield of three species of *Nectandra*

**DOI:** 10.1371/journal.pone.0204132

**Published:** 2018-09-18

**Authors:** Elza de Oliveira Ferraz, Maria Aparecida Ribeiro Vieira, Maria Izabela Ferreira, Ary Fernandes Junior, Márcia Ortiz Mayo Marques, Igor Otavio Minatel, Mariana Albano, Paolo Sambo, Giuseppina Pace Pereira Lima

**Affiliations:** 1 Department of Chemistry and Biochemistry, Institute of Biosciences, São Paulo State University (UNESP), Botucatu, São Paulo, Brazil; 2 Agronomic Institute, Campinas, Brazil; 3 Department of Microbiology and Immunology, Institute of Biosciences, São Paulo State University (UNESP), Botucatu, São Paulo, Brazil; 4 Department of Agronomy, Food, Natural Resources, Animals and the Environment, University of Padova, Padova, Italy; Tallinn University of Technology, ESTONIA

## Abstract

In this study, we aimed to determine whether seasonality affects the content, chemical composition, and antimicrobial activity of essential oils (EOs) from the leaves of three species of *Nectandra* (*Nectandra megapotamica*, *Nectandra grandiflora*, and *Nectandra lanceolata*) native to the Atlantic rainforest, Sao Paulo state, Brazil. In addition, we identified the compounds potentially related to the antimicrobial activity. Leaves were randomly collected in the middle of winter (August), spring (November), summer (February), and autumn (May). The influence of seasonality on the content and chemical composition of EOs from the *Nectandra* species was evident in this study. The EOs from *N*. *lanceolata* and *N*. *grandiflora* were characterized by similarities in the chemical composition and had a higher relative proportion of oxygenated sesquiterpenes. *N*. *megapotamica* presented a different chemical profile, with plenty of monoterpenic and sesquiterpenic hydrocarbons. Changes in the EO chemical profile because of seasonality were shown by the similarities between the EOs obtained in spring and autumn and the differences between the EOs obtained in summer and winter. The EO from the leaves of *N*. *megapotamica* harvested in winter and spring showed the highest control of the growth of *Escherichia coli*, and this antimicrobial action can be related to the monoterpenes α-pinene and β-pinene as well as myrcene and limonene. The minimum inhibitory concentration (MIC) of the EO from the leaves of *N*. *lanceolata* harvested in summer and autumn was lower against the gram-positive bacterium *Staphylococcus aureus* and can be related to the sesquiterpene hydrocarbons isobicyclogermacrenal, epi-zizanone, and germacrene B.

## Introduction

The genus *Nectandra* (family, Lauraceae) has a great diversity of essential oil (EO) constituents that may be influenced by seasonality; biome diversity; and plant age, part, and developmental stage [1,2]. Several environmental and climatic factors, such as atmospheric pollution, solar irradiation, soil, and water availability, as well as factors inherent to the plant may induce variations in the composition of secondary metabolites [3]. Thus, all these factors associated with harvest time are important and should be considered when assessing active plant compounds. For some species, such as *Laurus novocanariensis*, the EO yield ranging between 0.3 and 0.4% (v/w), this is, were nearly identical in the leaves collected during spring and autumn, respectively [4], whereas for *Aniba canelilla*, the contents of the main compounds (methyl eugenol and 1-nitro-2-phenylethane) were variable according to the season [3].

Species that belong to the family Lauraceae have confirmed antimicrobial activity, and the EO antifungal activity of some species, such as *Cinnamomum zeylanicum*, was high against several strains. However, *Aniba rosaeodora* and *Sassafras albidum* showed lower inhibitory activity [5]. Low EO concentrations (0.12% to 0.25%) from *A*. *rosaeodora* had potential antibacterial effects on *Escherichia coli* and *Staphylococcus aureus* [6]. EOs from other Lauraceae species (*Lindera pulcherrima*, *Dodecadenia grandiflora*, and *Persea gamblei)* were also studied, and they showed antibacterial effects against *S*. *aureus* and *Pasteurella multocida*; however, only the EO from *Persea gamblei* proved to be efficient against *E*. *coli* [7]. Therefore, in this study, we aimed to determine whether the yield, chemical composition, and antibacterial activity of EOs from *Nectandra megapotamica*, *Nectandra grandiflora*, and *Nectandra lanceolata* leaves are dependent on seasonal variations. In addition, we verified the chemical similarities of the EO by performing multivariate analysis, to identify compounds potentially related to antibacterial activity.

## Materials and methods

Fresh leaves of *N*. *megapotamica* (Spreng.) Mez, *N*. *grandiflora* Nees & Mart. ex Nees, and *N*. *lanceolata* were harvested from the experimental farm of Sao Paulo State University (latitude, 22° 50′ 48″ S; longitude, 48° 26′ 06″ W; and altitude, 817.74 m). The period of the experiment was characterized by high average temperatures (20–24°C) and significant precipitation in spring and summer (±132 mm). For sampling, we considered four plants of each species, and the leaves were harvested in the middle of each season, corresponding to winter (August), spring (November), summer (February), and autumn (May). The harvest was performed randomly for each plant (about 1.5 kg), and well-developed leaves (expanded and green) were selected. After the harvest, the material was homogenized, dried, and manually ground. Voucher specimens of *N*. *megapotamica* (30.066), *N*. *grandiflora* (30.011), and *N*. *lanceolata* (30.067) were deposited in the Herbarium “Irina Delanova Gemtchüjnicov”–BOTU, Department of Botany, Institute of Biosciences, UNESP, Botucatu, São Paulo, Brazil.

### EO extraction and analysis

The extraction of EOs from dried leaves (100 g) was performed by hydro-distillation with a Clevenger apparatus for approximately 2 h. For calculating EO yield, the extractions were performed in triplicate. The EO yield was 0.2 ± 0.03% (w/v), and the EOs were stored in amber glass bottles at 0°C.

Quantitative analyses (area standardization method) of the EOs were performed gas chromatography with flame ionization detection (GC–FID) and the chemical composition by gas chromatography coupled to mass spectrometer (GC-MS, Shimadzu, model QP-5000, Kyoto, Japan) equipped with a silica capillary column DB-5 (J&W Scientific, 30 m × 0.25 mm × 0.25 μm). Purified helium was used as the carrier gas at a flow rate of 1 mL min^-1^, and the injector and detector temperatures were 230°C and 240°C, respectively. The initial temperature of the oven was 60°C, and it was increased at 3°C/min up to 240°C. The EO (1 μL) was diluted in 1 mL of ethyl acetate (Tedia Brazil), and 1 μL of the mixture was injected in the split mode (1:20). The EO qualitative analyses were performed using a gas chromatograph connected to a mass spectrometer (GC-MS; Shimadzu, model QP-5000) operating by electron impact (70 eV), in scanning mode, at 1 scan/s, with a range of mass acquisition of 40–500 m/z, and interface temperature of 230°C. The chromatographic conditions were the same as those used in the quantitative analyses. EO components were identified by comparing their mass specters with literature data [8], the system’s GC/EM library (Wiley 139.Lib), and the retention rates determined by injecting n-alkanes C_9_–C_24_ (99%; Sigma Aldrich) under the same chromatographic conditions as that of the samples and applying the Van den Dool and Kratz equation [9].

### Antibacterial activity test

The standard strains of gram-negative (*Escherichia coli*, ATCC 43895) and gram-positive (*Staphylococcus aureus*, ATCC 25923) bacteria were grown on brain heart infusion agar plates at 37°C for 24 h under an aerobic environment. Then, inocula from each strain were prepared in Mueller-Hinton broth at 0.5 on the McFarland scale to obtaining concentrations of around 10^6^ colony-forming units (CFU)/mL. To assess the antibacterial activity of the EOs, viable cells were determined using the resazurin-based assay [10]. Two-hundred microliters of each inoculum was added to 96-well plates to verify the minimum inhibitory concentration (MIC). The EOs from each species were added to the wells to reach the following concentrations: 0.5% to 10.5% (v/v) for the tests with *E*. *coli* and 0.025% to 3% (v/v) for *S*. *aureus*. After inoculation, the microplates were incubated (37°C/24 h), and bacterial growth was measured after the addition of 50 μL of 0.01% resazurin, an oxidation-reduction indicator. The tests were performed in triplicate, and the MIC was confirmed as the lowest concentration without bacterial growth.

### Statistical analysis

The EO data were subjected to analysis of variance, and the means were compared using Tukey’s test (p ≤ 0.05) in the software GENES. Multivariate analysis of the relative average proportions of the compounds was performed using the principal component analysis (PCA) and hierarchical agglomerative clustering (HAC) in the XLSTAT (2012) program.

## Results and discussion

### Yield of EOs from *Nectandra* species

**Fig 1:**
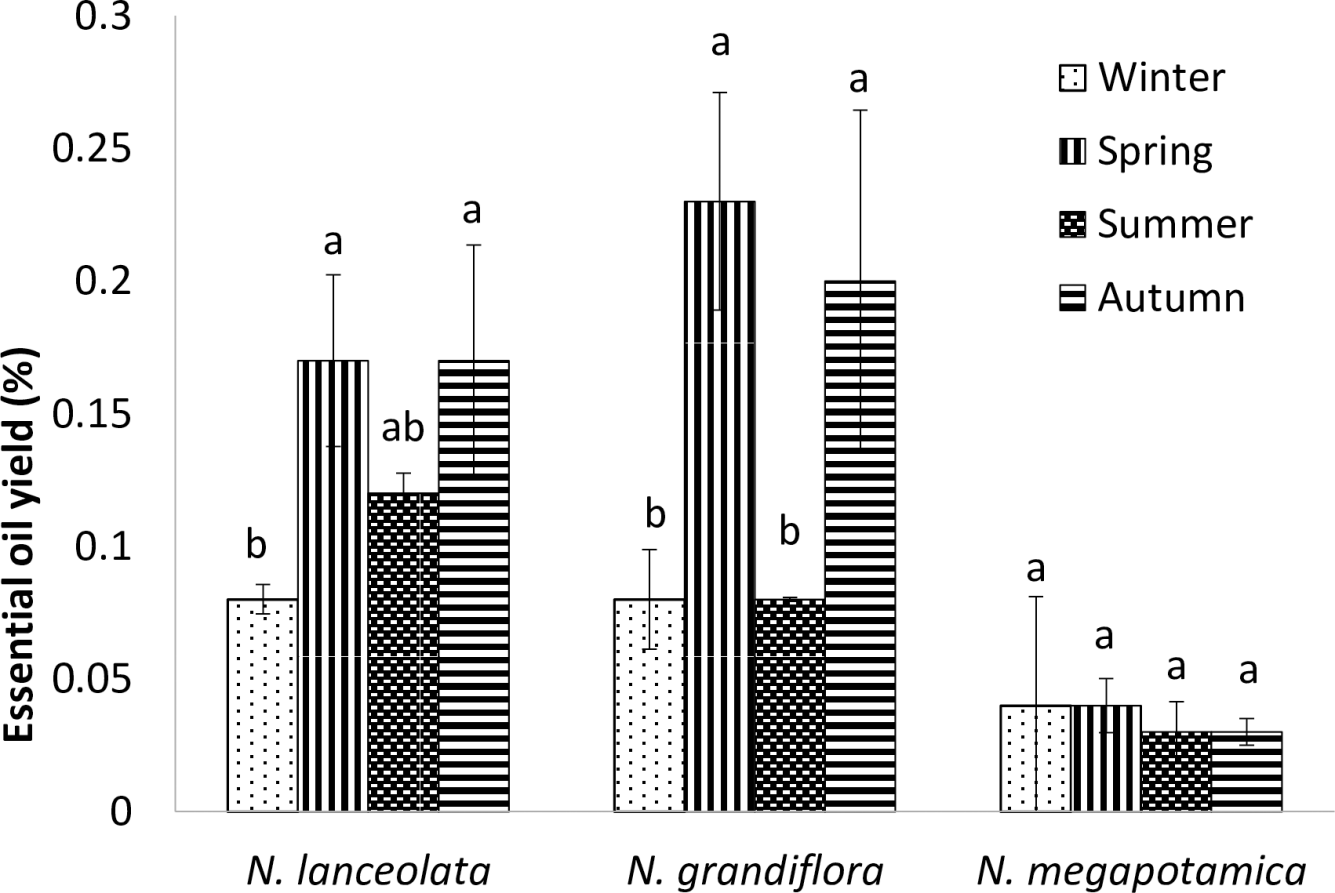
Essential oil yield (%) from the *Nectandra* species in different seasons (winter/2012, spring/2012, summer/2013, and autumn/2013). * Mean ± SD. Different letters are significantly different (p < 0.05).

The EO contents of the *N*. *lanceolata* and *N*. *grandiflora* species differed with the seasons. However, the EO content from *N*. *megapotamica* did not show significant variations over the seasons and showed the lowest yield (0.036 ± 0.08%). The EO yields of *N*. *grandiflora* and *N*. *lanceolata* were higher in spring and autumn, and these species showed their lowest yield in winter (Fig 1). The EO yield of *N*. *grandiflora* in spring (0.23%) did not differ from that in autumn (0.2%); however, it was higher than the EO yield in winter (0.08%), which is a season of low relative humidity. For *N*. *lanceolata*, the highest yield was obtained in spring and autumn (0.17%), without significant variations between these seasons.

The seasonality and phenological stage effects did not occur separately because the climatic and environmental factors induce changes in the plant phenology. Thus, the distinction between the phenological and seasonal effects becomes more complex. Both seasonality and phenology can influence the EO yield because the leaves show morphological and metabolic modifications [11]. In this study, we observed that the period of flowering occurred almost throughout the year, except in autumn when the plants were in the vegetative stage, as also described [12].

Our production results differ from the data found in the literature, and these variations could be attributed to the specific climatic conditions of the studied region. High EO yields (0.45% and 0.33%) were found in *N*. *megapotamica* harvested in spring and summer, respectively [11]; however, higher EO yields were obtained from *N*. *grandiflora* (0.75%) harvested in spring [13]. In our study, we did not observe significant differences among the seasons with respect to the EO content of *N*. *megapotamica*; however, we found higher EO contents in autumn and spring than in winter and autumn for *N*. *lanceolata* and *N*. *grandiflora*. Thus, to achieve higher EO yields, the harvest of the leaves from these two species should be performed in autumn, when the plants are in their vegetative stage, because the leaves harvested during the reproductive period (spring) can show alterations due to pollination and fructification. Another important factor for determining the best time for harvesting the leaves is the phytochemical profile of the EOs.

### Chemical composition of the EOs

In all the EOs from the studied species of *Nectandra*, we identified substances from the classes of monoterpenic hydrocarbons, oxygenated monoterpenes, sesquiterpenic hydrocarbons, and oxygenated sesquiterpenes. The chemical compositions of EOs from *N*. *grandiflora* and *N*. *lanceolata* were similar; both had the highest relative proportion of substances from the class of oxygenated sesquiterpenes. However, the EO profile of *N*. *megapotamica* showed more substances from the classes of monoterpenic and sesquiterpenic hydrocarbons (Table 1).

**Table 1:** Relative percentage of EO components in the *Nectandra* species in different seasons of the year (winter/2012, spring/2012, summer/2013, and autumn/2013).

Substances	RI^a^	*N*. *grandiflora*				*N*. *lanceolata*				*N*. *megapotamica*			
		W	Sp	Su	A	W	Sp	Su	A	W	Sp	Su	A
**α-pinene**	930	0.6	0.3	0.1	0.1	1.4	1.0	0.1	0.6	20.1	18.2	10.1	25.1
**camphene**	945	−	−	−	−	0.2	0.1	0.1	0.1	0.8	0.6	0.3	0.9
**sabinene**	970	−	−	−	−	0.1	0.1	0.1	0.1	−	−	−	−
**β-pinene**	974	0.4	0.2	−	0.3	1.3	0.9	0.1	0.1	18.5	16.2	9.6	22.3
**myrcene**	987	−	−	−	−	−	−	−	−	1.1	1.6	1.3	1.4
**α-phellandrene**	1003	−	−	−	−	−	−	−	−	0.4	10.0	11.0	0.3
**limonene**	1025	0.4	0.3	0.1	0.3	0.4	0.3	0.2	0.2	5.8	7.3	5.5	7.7
**1,8-cineole**	1028	−	−	−	−	−	−	−	−	1.1	1.9	1.3	1.3
***Z*-β-ocimene**	1034	−	−	−	−	0.1	0.3	0.2	0.2	−	−	−	−
**(*E*)-β-ocimene**	1044	0.1	0.2	0.1	0.7	0.7	1.8	1.3	1.7	0.8	1.2	0.7	2.5
**terpinolene**	1085	−	−	−	−	−	−	−	−	0.2	0.3	0.1	0.1
**linalool**	1097	0.4	0.4	0.4	0.1	3.3	0.7	1.9	1.0	−	−	−	−
**terpinen-4-ol**	1174	−	−	−	−	−	−	−	−	0.1	0.3	0.2	0.3
***cis-*3-hexenyl butanoate**	1183	0.2	0.2	0.2	0.3	0.2	0.1	0.3	0.2	0.1	−	−	0.3
**(*Z*)-3-hexenyl 2-methyl butanoate**	1230	−	−	−	−	−	−	−	−	0.1	0.3	0.1	0.1
**isobornyl acetate**	1283	−	−	−	−	0.2	−	0.1	0.1	−	−	−	−
**δ-elemene**	1336	0.4	0.9	0.6	1.5	−	−	−	−	0.4	0.3	0.1	0.3
**α-cubebene**	1348	−	−	−	−	−	−	−	−	4.2	3.5	5.8	3.6
**α-copaene**	1374	−	−	−	−	−	−	−	−	3.6	2.0	2.7	1.8
**β-bourbonene**	1383	−	−	−	−	−	−	−	−	0.1	0.1	0.2	0.2
**β-cubebene**	1388	−	−	−	−	−	−	−	−	1.8	1.3	1.9	0.9
**β-elemene**	1390	0.2	0.3	0.1	0.4	0.2	−	−	−	0.1	−	−	−
***trans*-caryophyllene**	1417	0.7	0.9	0.1	0.9	−	−	−	−	5.6	4.1	4.6	3.0
**α-humulene**	1451	0.2	0.1	0.1	0.1	−	−	−	−	2.1	1.6	2.2	1.3
***allo*-aromadendrene**	1458	−	−	−	−	−	−	−	−	0.7	0.7	1.1	0.1
**γ-murolene**	1474	−	−	−	−	−	−	−	−	0.6	0.1	0.7	0.1
**germacrene D**	1479	0.71	0.10	0.24	0.21	0.52	0.87	0.52	0.51	7.25	5.16	6.73	3.66
**β-selinene**	1484	2.8	2.6	3.0	2.3	3.9	5.6	3.5	3.1	−	−	−	−
**bicyclogermacrene**	1494	1.1	2.9	0.8	4.2	5.2	12.6	4.8	5.5	10.6	8.7	14.8	9.1
**α-muurolene**	1498	−	−	−	−	−	−	−	−	0.2	0.1	0.2	−
**germacrene A**	1502	−	−	−	0.1	−	−	−	−	−	−	−	−
**δ-amorphene**	1512	−	−	−	−	−	−	−	−	0.7	0.7	1.0	0.9
**7-*epi-*α-selinene**	1514	0.1	0.1	−	0.1	−	−	−	−	−	−	−	−
**δ-cadinene**	1520	−	−	−	−	−	−	−	−	2.7	1.7	1.9	1.3
**elemol**	1546	0.3	0.3	0.3	0.3	0.2	0.2	0.1	0.1	−	−	−	−
**germacrene B**	1554	−	−	−	−	0.1	0.3	0.3	0.4	−	−	−	−
**spathulenol**	1574	20.1	13.3	18.5	11.1	20.2	7.6	15.9	11.9	3.2	4.1	5.5	4.1
**caryophyllene oxide**	1580	3.3	1.9	4.4	1.5	1.8	1.0	1.5	1.7	1.2	1.4	1.7	1.3
**1-*epi*-cubenol**	1625	−	−	−	−	−	−	−	−	0.1	0.1	0.1	0,2
**muurola-4,10(14)-dien-1-β-ol**	1629	2.8	3.3	1.9	3.2	−	−	−	−	−	−	−	−
**cubenol**	1638	−	−	−	−	−	−	−	−	0.2	0.2	0.4	0.3
**α-muurolol**	1642	−	−	−	−	−	−	−	−	0.1	0.1	0.2	0.2
**β-eudesmol**	1646	1.0	0.8	1.4	0.9	−	−	−	−	0.2	0.1	0.3	0.3
**α-eudesmol**	1650	0.2	0.1	0.3	0.1	−	−	−	−	−	−	−	−
***epi-*zizanone**	1664	3.0	3.1	3.2	3.0	1.8	2.1	2.4	2.6	−	−	−	−
**isobicyclogermacrenal**	1729	29.6	39.1	27.8	39.6	30.0	34.1	34.3	41.8	−	−	−	−
**rosadiene**	1932	15.1	11.6	16.6	11.2	6.1	3.9	4.5	3.6	−	−	−	−
**kaurene**	2031	5.0	3.9	4.3	2.2	3.1	2.7	2.5	1.1	−	−	−	−
**Monoterpene Hydrocarbons**	−	1.4	1.1	0.3	1.8	4.2	4.5	2.8	3.3	49.2	57.3	40.3	62
**Oxygenated Monoterpenes**	−	0.1	0.6	0.1	0.8	3.7	0.9	2.2	1.2	0.4	0.6	0.3	1.1
**Sesquiterpene Hydrocarbons**	−	6.2	7.9	5.4	9.7	10	19.4	9.1	9.5	40.6	30.4	44.2	27
**Oxygenated Sesquiterpenes**	−	60.7	62.2	57.7	60.1	53.8	44.9	54.2	58.2	5.0	6.0	8.2	6.4
**Other classes**	−	20.1	15.5	20.9	13.4	9.1	6.7	7.0	4.7	−	−	−	−
**Total identified**	−	80.0	87.3	84.8	85.9	80.8	76.3	75.4	76.9	95.1	94.3	93	96.5

W: winter, Sp: spring, Su: summer, A: autumn; RI^a^: retention index obtained using an DB-5 column.— = Trace (<0.1%).

The major compounds in both *N*. *grandiflora* and *N*. *lanceolata* were isobicyclogermacrenal, spathatulenol, and rosadiene, whereas the major compounds in *N*. *megapotamica* were α-pinene, β-pinene, and bicyclogermacrene. We verified that both classes and major compounds showed alterations in function with seasonality in the three species. The oxygenated sesquiterpenes occurred at higher levels in spring (62.2%) in *N*. *grandiflora* and in autumn (58.2%) in *N*. *lanceolata*. In *N*. *grandiflora*, isobicyclogermacrenal was the major compound observed in autumn and spring (39.6% and 39.1%), spathulenol in winter (20.1%), and rosadiene in summer (16.6%). *N*. *lanceolata* showed high values of isobicyclogermacrenal in autumn (41.8%), and the highest contents of both spathulenol (20.2%) and rosadiene (6.1%) were observed in winter (Table 1).

The main compounds of *N*. *megapotamica* also showed alterations with seasonality. The highest content of monoterpenic hydrocarbons was observed in autumn (62.2%), and the highest content of sesquiterpenic hydrocarbons was found in summer (44.2%; Table 1). In this species, we also observed that the contents of α-pinene (25.1%) and β-pinene (22.3%) were the highest in autumn and bicyclogermacrene content increased in summer (14.8%; Table 1).

Besides the quantitative variations in the main components, small variations were found in most of the EO compounds in each species; qualitative variations, such as the presence or absence of a certain compound in different seasons, were also observed (Table 1). In this study, a wide variation in the relative percentage of α-phellandrene was detected; this compound could be considered a major compound in spring (10%) and summer (11%). However, this result was not repeated in winter and autumn because the values were very low (0.4% and 0.3%, respectively; Table 1).

### Antibacterial activity

EOs from the three *Nectandra* species showed antibacterial activity against both *E*. *coli* and *S*. *aureus*. We observed that *E*. *coli* was more vulnerable to the EOs from *N*. *megapotamica*, whereas *S*. *aureus* was more susceptible to the EOs from *N*. *lanceolata*, regardless of the season of the year in which the leaves were harvested (Table 2). The lowest MIC for *E*. *coli* was observed when the bacteria were exposed to the EOs from the leaves of *N*. *megapotamica* harvested in winter.

**Table 2:** Minimum inhibitory concentration (MIC, %) of essential oils from the leaves of *Nectandra lanceolata*, *N*. *megapotamica*, and *N*. *grandiflora*, harvested in all seasons, against *E*. *coli* and *S*. *aureus* ATCC strains.

	Winter	Spring	Summer	Autumn
	***E*. *coli***			
***N*. *lanceolata***	7.50±1.44*	4.00±0.58	10.10±0.02	10.10±0.07
***N*. *megapotamica***	2.25±0.75	5.50±0.58	6.50±1.02	6.75±1.89
***N*. *grandiflora***	6.50±2.02	4.25±0.96	10.10±0.03	10.10±0.02
	***S*. *aureus***			
***N*. *lanceolata***	0.60±0.04	0.70±0.17	0.55±0.05	0.55±0.05
***N*. *megapotamica***	1.05±0.43	1.90±0.64	1.90±0.64	3.00±0.02
***N*. *grandiflora***	1.90±0.64	1.80±0.69	1.90±0.64	3.00±0.08

*Means ± SD.

The inhibitory action against bacterial growth can be attributed to the effects of the secondary compounds, which have shown higher inhibitory action against gram-positive bacteria than against gram-negative ones and yeast [14]. This is because the gram-negative bacteria have a diversified cell wall, with an external membrane composed specially by lipopolysaccharides, granting higher resistance by avoiding the diffusion and accumulation of EOs in the bacterial cell [15]. The antimicrobial effects generally occur because of structural and functional damage to the plasmatic membrane, mainly due to the hydrophobicity of the compounds present in EOs [16], as well as the influence on the enzymatic systems and genetic material of the bacteria [17,18]. Thus, qualitative analyses of EOs can reveal potentially important substances for bacterial control.

### Chemical similarities

**Fig 2:**
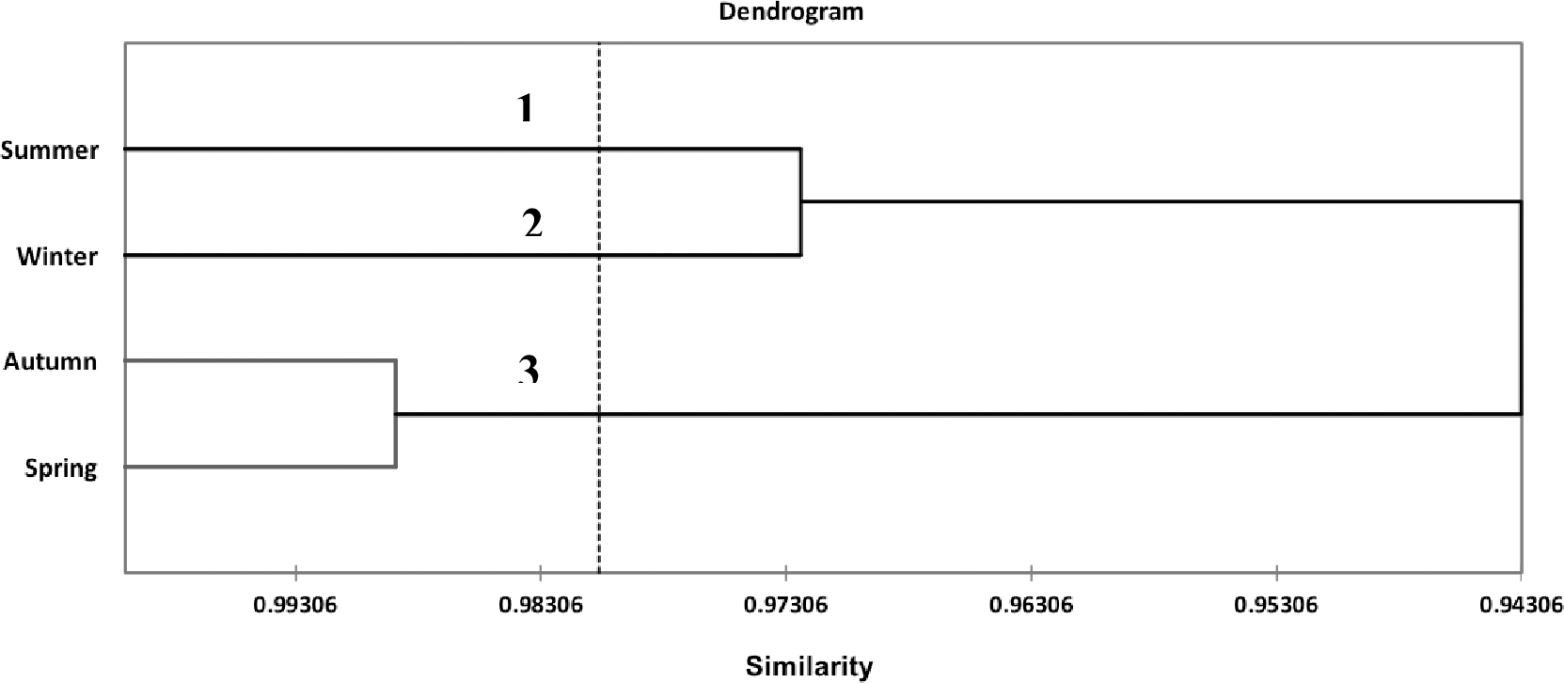
Dendrogram obtained by hierarchical agglomerative clustering (HAC) analysis of the substances identified in the essential oils (EOs) obtained from the three *Nectandra* species during the four seasons of the year.

The chemical similarities among the EOs extracted from the leaves harvested in different seasons were studied by performing multivariate analysis of the chemical compounds, regardless of the species. The Hierarchical Agglomerative Cluster (HAC) analysis (Fig 2) showed the formation of three classes for the seasons, regardless of the species. Class 1 is formed by the EO obtained in summer; class 2, winter; and class 3, spring and autumn, demonstrating the chemical similarities between these two seasons.

**Fig 3:**
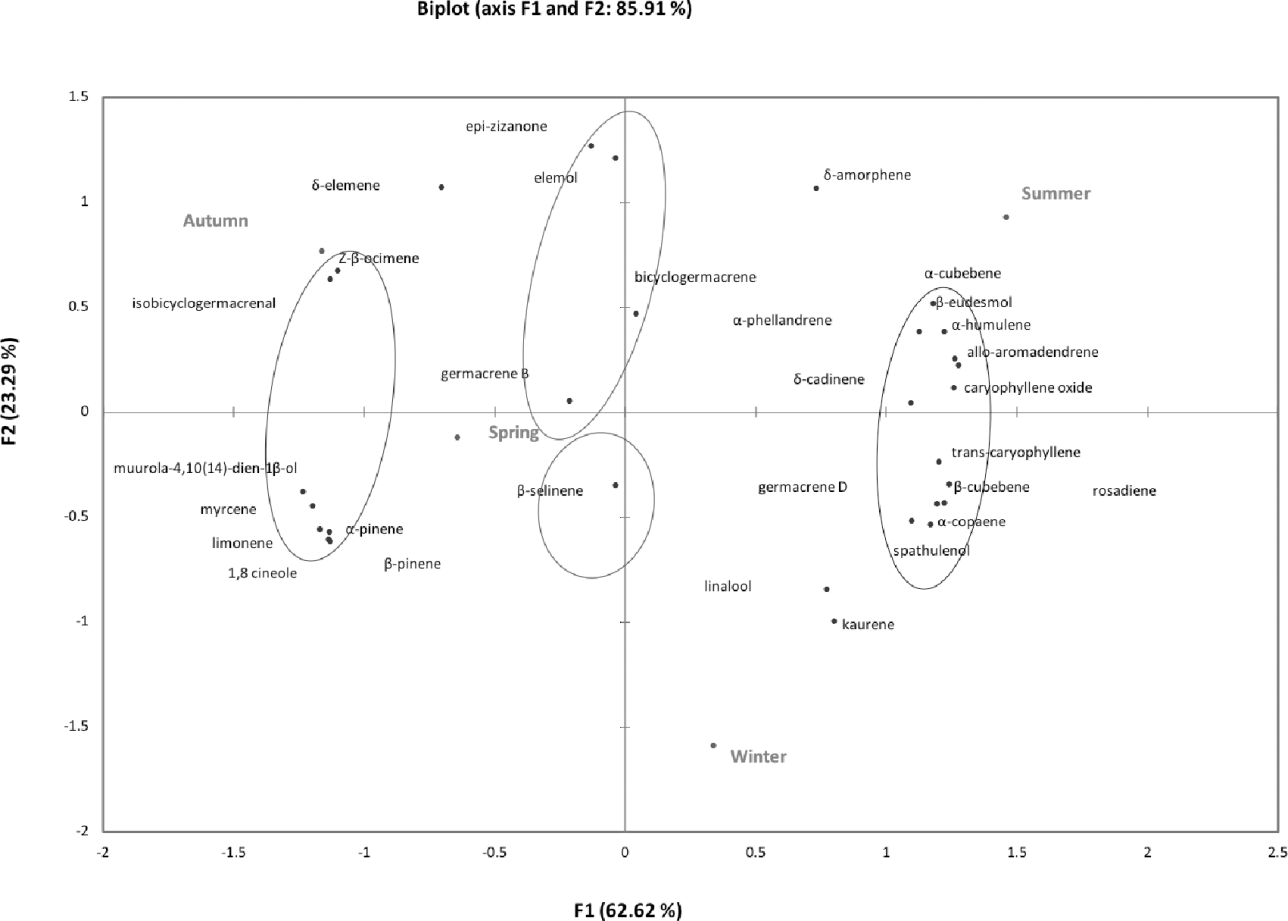
Principal component analysis (PCA) of the substances identified in the essential oils (EOs) obtained from the three *Nectandra* species during the four seasons of the year.

PC1 and PC2 explained 85.91% of the data variance (F1 and F2) (Fig 3). The PCA of EOs in the different seasons of the year showed that summer (class 1) shares many substances with winter (class 2), but these two seasons are located far from each other. Autumn and spring (class 3) are closer and share many compounds.

Some substances were shared between autumn and summer as well as between spring and winter, which shows the chemical similarities between the EOs under the seasonality effect. On the basis of HAC (Fig 2) and PCA (Fig 3), we can affirm that the high similarity observed in class 3 (spring and autumn) is due to the compounds shared between these two seasons, and, at the same time, these compounds are different from those in classes 1 and 2 (summer and winter, respectively), which also share many compounds.

We can also identify some compounds for each season of the year. δ-amorphene, α-cubebene, α-phellandrene, β-eudesmol, allo-aromadendrene, α-humulene, caryophyllene oxide, and δ-cadinene are related to summer. Trans-caryophyllene, β-cubebene, rosadiene, germacrene D, spathulenol and α-copaene are related to winter; muurola-4,10(14)-dien-1β-ol, myrcene, 1,8-cineole, limonene, α-pinene, β-pinene, and β-selinene are related to spring. Germacrene B, δ-elemene, epi-zizanone, and elemol are related to autumn; Z-β-ocimene and isobicyclogermacrenal are closely related.

**Fig 4:**
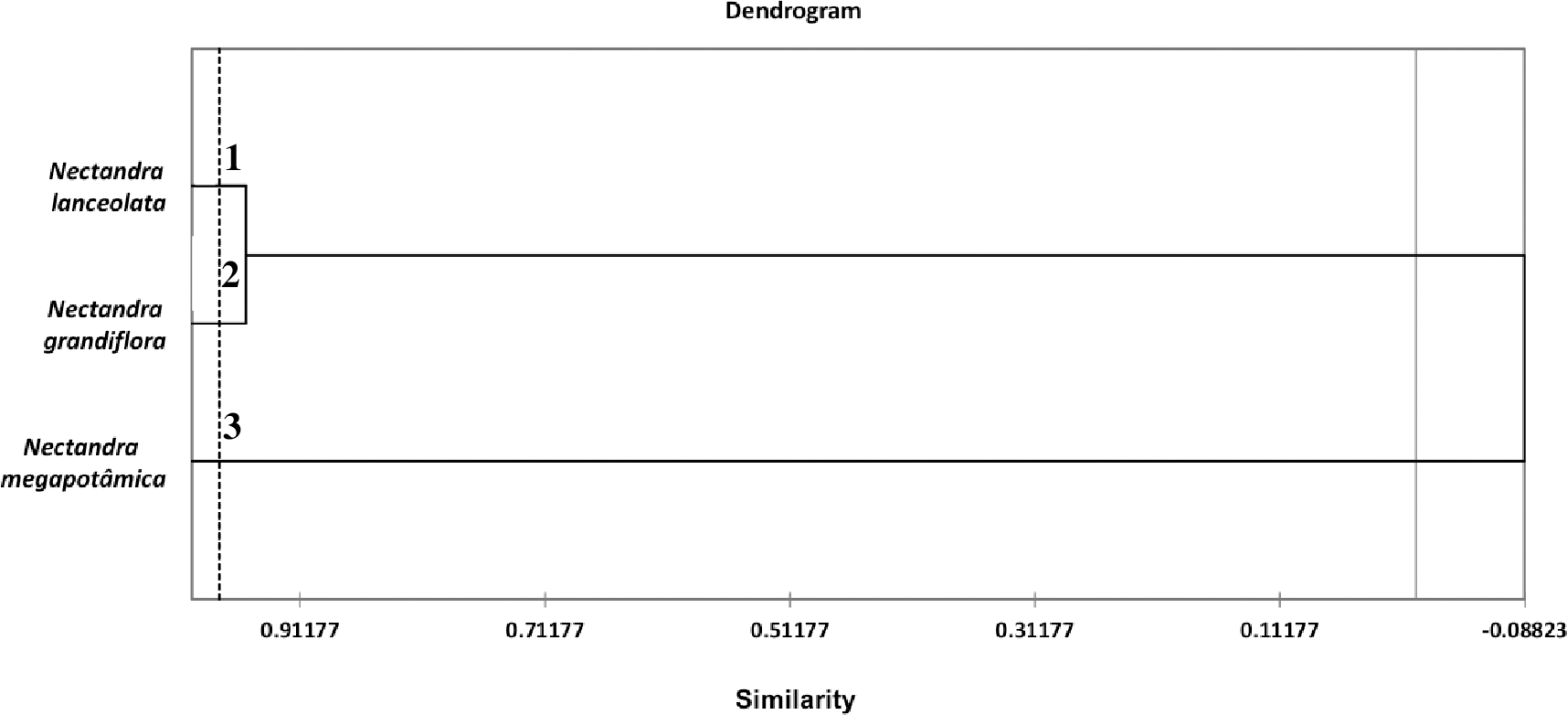
Dendrogram of the hierarchical agglomerative clustering (HAC) analysis of the substances identified in the essential oils (EOs) obtained from the three *Nectandra* species during the four seasons of the year.

**Fig 5:**
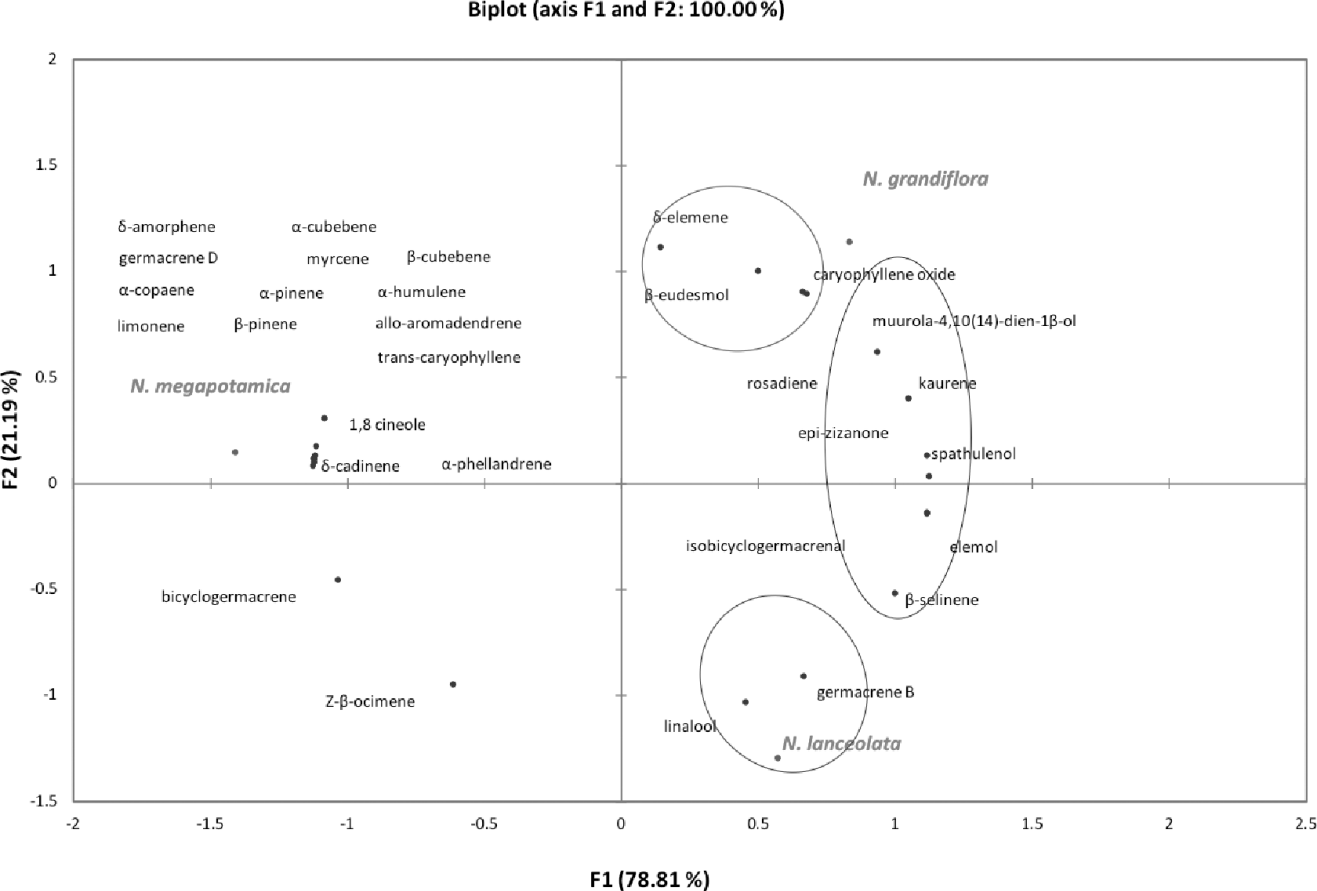
Principal component analysis (PCA) of the substances identified in the essential oils (EOs) obtained from the three *Nectandra* species during the four seasons of the year.

The similarities in the chemical composition of EOs from the three *Nectandra* species harvested in different seasons of the year are shown in Fig 4. According to HAC, the species formed three distinct classes. Class 1 comprises *N*. *lanceolata*; class 2, *N*. *grandiflora*; and class 3, *N*. *megapotamica*; however, class 3 is chemically distinct from the other ones (Fig 4). The PCA confirms the similarities between *N*. *lanceolata* and *N*. *grandiflora* as well as the separation of *N*. *megapotamica* because it showed 100% data variations in two components (F1 and F2) (Fig 5). The PCA showed that *N*. *megapotamica* contains limonene, 1,8-cineole, α-pinene, β-pinene, α-phellandrene, α-cubebene, β-cubebene, α-copaene, trans-caryophyllene, α-humulene, δ-amorphene, allo-aromadendrene, δ-cadinene, and germacrene D, and these compounds differentiate this species from the others. The compounds shared by *N*. *grandiflora* and *N*. *lanceolata* are elemol, isobicyclogermacrenal, spathulenol, β-selinene, epi-zizanone, and kaurene. The compounds related to *N*. *lanceolata* are germacrene B and linalool, and those related to *N*. *grandiflora* are δ-elemene, β-eudesmol, caryophyllene oxide, and muurola-4,10(14)-dien-1β-ol.

**Fig 6:**
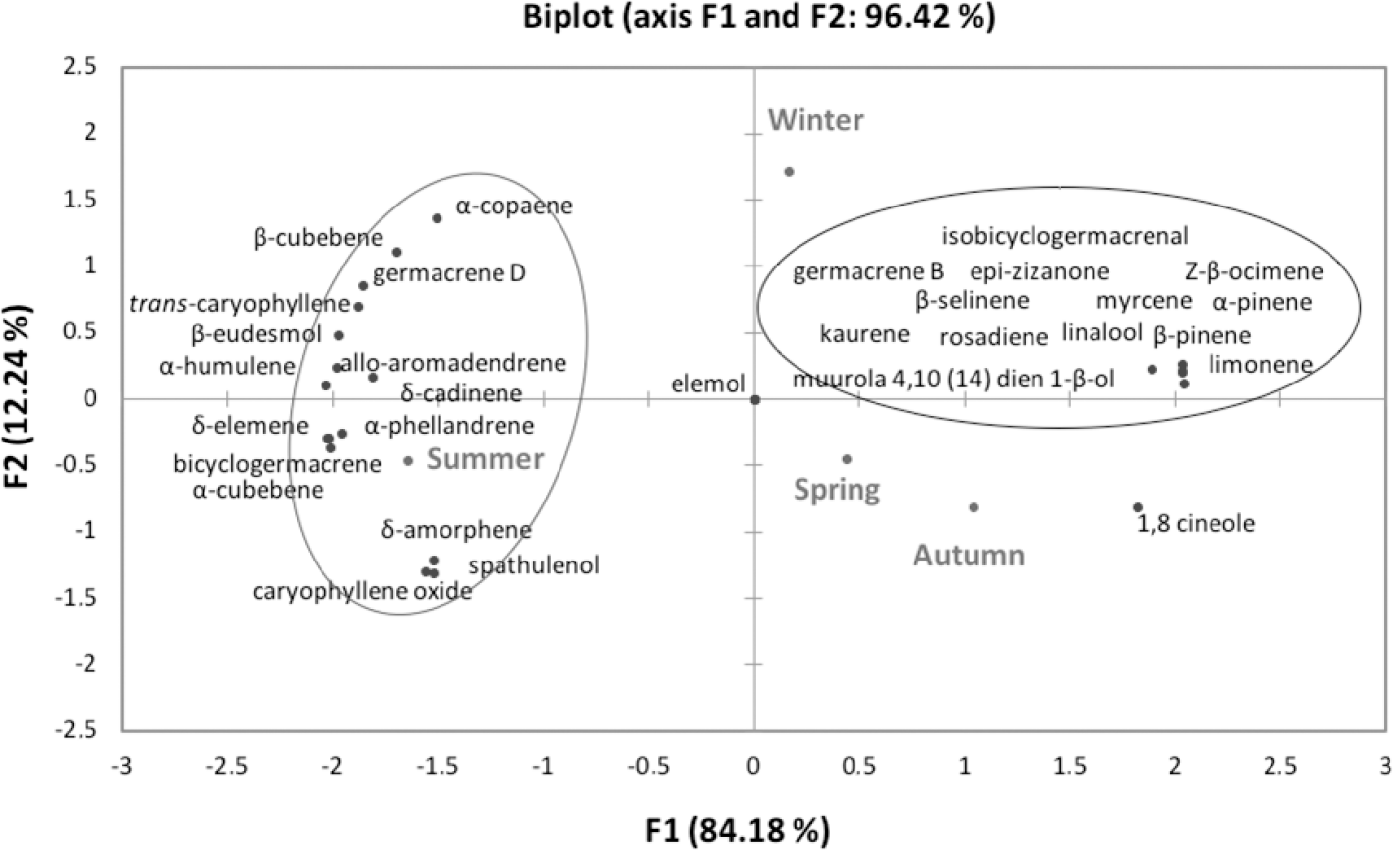
Principal component analysis (PCA) of the substances identified in the essential oils (EOs) obtained from the three *Nectandra* species during the four seasons of the year.

To investigate the substances possibly responsible for the antimicrobial activity of *N*. *megapotamica* against *E*. *coli* and of *N*. *lanceolata* against *S*. *aureus*, we studied the chemical composition of the EOs in different seasons of the year by using PCA. Fig 6 shows the PCA of the EO compounds from *N*. *megapotamica* in different seasons, which expresses 96.42% of the data variations in the two components. We found that elemol is a common substance in all the seasons, located in the center of the graphic. Summer is located far from the other seasons, which are also far from the grouped compounds; this indicates differentiation in the chemical composition of the EOs collected in this season. However, we observed that among the compounds related to winter, spring, and autumn, the monoterpenes α-pinene and β-pinene are the major compounds of *N*. *megapotamica* (Table 1). Myrcene and limonene showed similarities in their phytochemical profiles between seasons and the highest relative proportions during winter, spring, and autumn.

**Fig 7:**
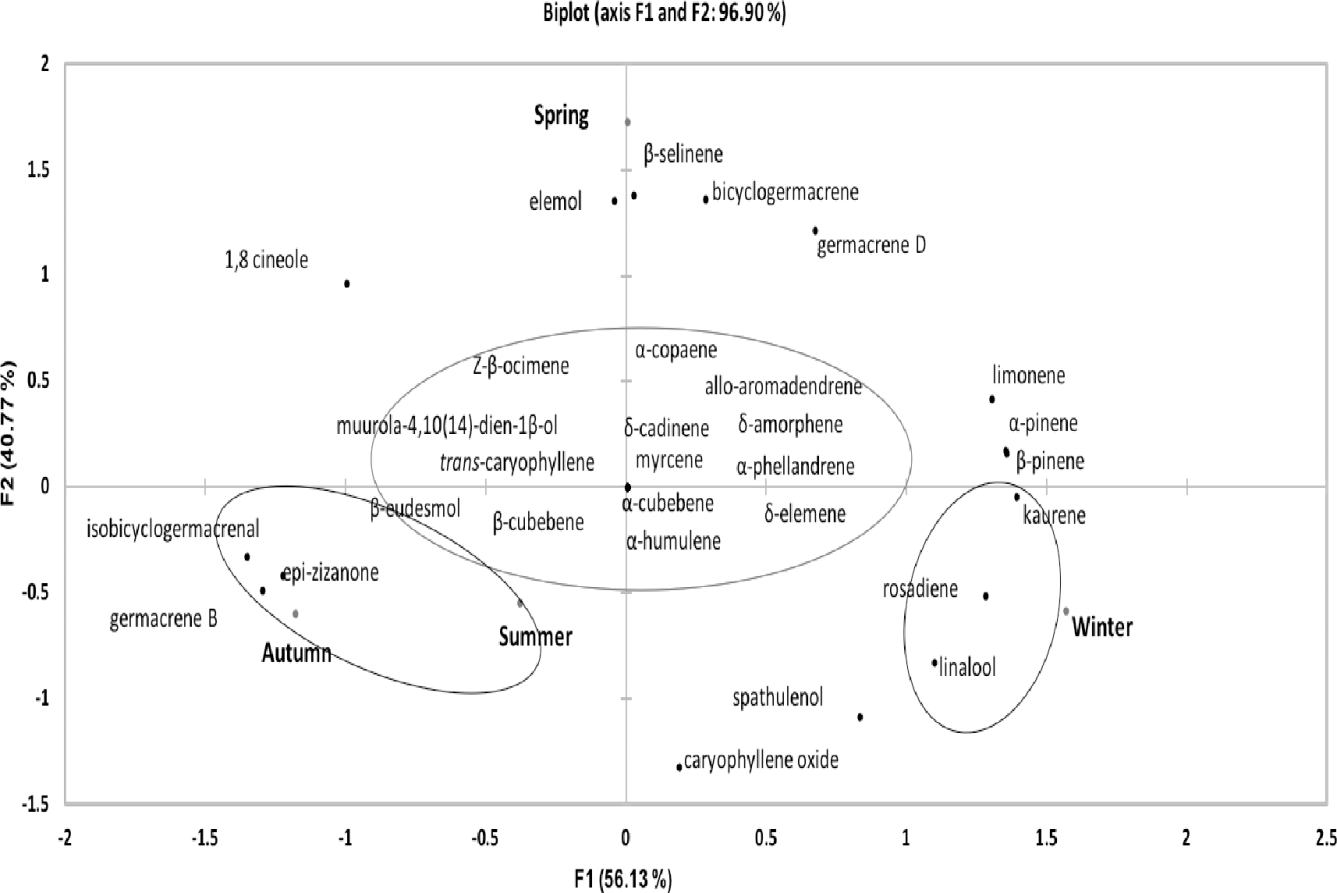
Principal component analysis (PCA) of the substances identified in the essential oils (EOs) obtained from the three *Nectandra* species during the four seasons of the year.

Although PCA shows clear differences between summer and the other seasons, the influence of the monoterpenes on the antimicrobial activity is unclear because a lower MIC (Table 2) of the EOs from *N*. *megapotamica* against the gram-negative bacteria (*E*. *coli*) was observed in winter, the season that did not show a distinct profile in the PCA. However, different results were observed for the EOs from *N*. *lanceolata* in different seasons, which express 96.90% of the data variations in the two components (Fig 7). We observed many compounds that were common for the seasons, located in the center of Fig 7 and the differentiating substances for spring, winter, autumn, and summer. Besides elemol, specific compounds, i.e., the sesquiterpenes β-selinene and bicyclogermacrene, were observed in spring (Fig 7; Table 1). The substances related to winter (Fig 7) were kaurene, rosadiene, and linalool, which also showed higher contents in this season (Table 1). For summer and autumn (Fig 7), the discriminating substances was the sesquiterpene hydrocarbon isobicyclogermacrenal; the major compounds were epi-zizanone and germacrene B, which showed a more significant increase in these seasons than in autumn (Table 1). In addition, we verified a lower MIC for *S*. *aureus* in these seasons (Table 2). Thus, these substances are possibly responsible for the high antimicrobial activity against the gram-positive bacteria. However, it is important to point out that the observed antimicrobial activity may not be directly related to the major compounds, but to the synergy between the compounds.

Some studies have emphasized that EOs containing oxygenated sesquiterpenes show antimicrobial activity, such as the *in vitro* study performed to determine the antimicrobial activity of EO from a species of *Ocimum* [19]. *Ocimum suave* collected from Tanzania had 14% germacrene B and a moderate antimicrobial activity against *S*. *aureus* (1.35 mg/mL) and *E*. *coli* (1.95 mg/mL). Germacrene B has already been evaluated separately [20]. In this study, EOs from the aerial parts of *Artemisia indica* (containing 8.6% germacrene B as the isolated compound) exhibited antimicrobial activity. This compound was screened *in vitro* and showed spectrum antimicrobial activity against the gram-positive bacterium *S*. *aureus* and especially against gram-negative bacteria [20].

## Conclusion

The seasonal variations had significant effects on the content and chemical composition of the EOs from the *Nectandra* species. The EO contents from *N*. *lanceolata* and *N*. *grandiflora* were higher in spring and autumn. These two species were also characterized by their similar EO chemical compositions, whereas *N*. *megapotamica* had a distinct chemical profile, constituted a separate class, and did not show variations in the EO content. Alterations to the EO chemical profile with seasonality were also observed, and class 1 was formed by the EO of leaves harvested in summer; class 2, EO of leaves collected in winter; class 3, EO of leaves harvested in spring and autumn. The EO of *N*. *megapotamica* collected in winter and spring showed the highest control of growth of *E*. *coli*. Thus, the action on gram-negative bacteria may be related to the monoterpenes α-pinene and β-pinene, which are the major compounds of the EO, besides myrcene and limonene, which had a relation to winter, autumn, and spring. *N*. *lanceolata* showed high antibacterial potential against the gram-positive bacterium *S*. *aureus*, and this bioactivity can be related to the sesquiterpene hydrocarbons isobicyclogermacrenal (major EO compound), epi-zizanone, and germacrene B, which showed an increase in summer and autumn (seasons when the EO showed a lower MIC). In the literature, the data for the biological activity of EOs from *Nectandra* species are limited, and, certainly, future studies should be performed to elucidate which compounds of the EOs from *N*. *megapotamica* and *N*. *lanceolata* are truly responsible for the growth of gram-negative and gram-positive bacteria, respectively.
